# Rapid Assays for Specific Detection of Fungi of *Scopulariopsis* and *Microascus* Genera and *Scopulariopsis brevicaulis* Species

**DOI:** 10.1007/s11046-016-0008-5

**Published:** 2016-06-02

**Authors:** Milena Kordalewska, Tomasz Jagielski, Anna Brillowska-Dąbrowska

**Affiliations:** Department of Molecular Biotechnology and Microbiology, Faculty of Chemistry, Gdańsk University of Technology, Narutowicza 11/12, 80-233 Gdańsk, Poland; Department of Applied Microbiology, Faculty of Biology, Institute of Microbiology, University of Warsaw, Miecznikowa 1, 02-096 Warsaw, Poland

**Keywords:** Detection, Identification, *Microascus*, PCR, Real-time PCR, *Scopulariopsis*

## Abstract

**Purpose:**

Fungi of *Scopulariopsis* and *Microascus* genera cause a wide range of infections, with *S. brevicaulis* being the most prevalent aetiological agent of mould onychomycosis. Proper identification of these pathogens requires sporulating culture, which considerably delays the diagnosis. So far, sequencing of rDNA regions of clinical isolates has produced ambiguous results due to the lack of reference sequences in publicly available databases. Thus, there is a clear need for the development of new molecular methods that would provide simple, rapid and highly specific identification of *Scopulariopsis* and *Microascus* species. The objective of this study was to develop simple and fast assays based on PCR and real-time PCR for specific detection of fungi from *Scopulariopsis* and *Microascus* genera, and separately, *S. brevicaulis* species.

**Methods:**

On the basis of alignment of β-tubulin gene sequences, *Microascus/Scopulariopsis*-specific primers were designed and *S. brevicaulis*-specific primers were reevaluated. DNA from cultured fungal isolates, extracted in a two-step procedure, was used in *Microascus/Scopulariopsis*-specific and *S. brevicaulis*-specific PCR and real-time PCR followed by electrophoresis or melting temperature analysis, respectively.

**Results:**

The specificity of the assays was confirmed, as positive results were obtained only for *Scopulariopsis* spp. and *Microascus* spp. isolates tested in *Microascus/Scopulariopsis*-specific assay, and only for *S. brevicaulis* and *S. koningii* (syn. *S. brevicaulis*) isolates in a *S. brevicaulis*-specific assay, respectively, and no positive results were obtained neither for other moulds, dermatophytes, yeast-like fungi, nor for human DNA.

**Conclusions:**

The developed assays enable fast and unambiguous identification of *Microascus* spp. and *Scopulariopsis* spp. pathogens.

## Introduction

The genus *Scopulariopsis*, erected by Bainier (1907), contains both hyaline and dematiaceous moulds, which propagate asexually by conidia. Most of their teleomorphs are included in the genus *Microascus* [1–5]. The anamorph–teleomorph connections have already been established for many species. However, the sexual states of some *Scopulariopsis* species are still unknown [6].

*Scopulariopsis* spp. are saprobes with a worldwide distribution. They are commonly isolated from soil, air, plant debris, paper, dung and moist indoor environments [7, 8]. Traditionally, *Scopulariopsis* and *Microascus* species have not been considered common human pathogens. However, the number of cases with these organisms as main perpetrators has recently been on the rise. Some species are known to be opportunistic pathogens, primarily causing superficial tissue infections, and being one of the principal causes of non-dermatophytic onychomycoses [9, 10]. The prevalence of onychomycosis caused by *S. brevicaulis* is estimated to make up 3–10 % of the total number of mould onychomycosis cases globally. Clinically, the condition is generally recognised as distal and lateral subungual onychomycosis (DLSO) [11, 12]. Cases of cutaneous and subcutaneous infections have also been described as due to *S. brevicaulis* [13, 14]. Less commonly *Scopulariopsis* and *Microascus* species have been reported as causes of other infections including endocarditis [15–18], keratitis [19, 20], endophthalmitis [21], sinusitis [22, 23], pulmonary fungus ball [24, 25], otomycosis [26, 27], pneumonia [28–30], peritonitis [31], cerebral phaeohyphomycosis and brain abscess [32–34], disseminated infection with skin lesions including a patient with acquired immune deficiency syndrome (AIDS) [13], disseminated infection after bone marrow transplantation [35, 36], invasive infection after lung [37, 38] or heart and lung transplantation [39].

Among *Scopulariopsis* and *Microascus* species most frequently isolated from all types of lesions, *S. brevicaulis* ranks first, followed by *S. acremonium*, *S. brumptii*, *S. flava*, *M. niger*, *M. cinereus*, *M. cirrosus*, *M. manginii*, and *M. trigonosporus* [6, 40].

The data considering *Scopulariopsis* and *Microascus* antifungal susceptibility are scarce and often inconsistent. The very few reports available have recognised them as a multidrug-resistant fungi [41, 42]. Noteworthy, the lack of correlation between in vitro drug susceptibility (MIC determination results) and clinical outcomes has been demonstrated [39, 41].

The recovery of *Scopulariopsis* and *Microascus* species from clinical samples is relatively easy, as these fungi grow well on routine laboratory media. Yet, it is still difficult to perform species identification based on morphological criteria. Moreover, *Microascus/Scopulariopsis* infections, and disseminated infections in particular might be clinically and histologically indistinguishable from aspergillosis, fusariosis or zygomycosis [43, 44]. Since, in the majority of clinical reports on *Scopulariopsis* spp. infections, morphological identification of the aetiological agent has not been confirmed at the molecular level, the actual prevalence of *Scopulariopsis* species, other than *S. brevicaulis*, is unknown [6].

In this paper, we present PCR and real-time PCR-based assays developed for the detection of cultured isolates of *Scopulariopsis* and *Microascus* genera, as well as *S. brevicaulis* species.

## Materials and Methods

### Strains and Isolates

In the present study, we used a total of 219 fungal strains, representing 103 fungal species (Table 1). The strains were obtained from international culture collections (CBS-KNAW Fungal Biodiversity Centre; BCCM/IHEM Biomedical Fungi and Yeasts Collection—Belgian Coordinated Collections of Microorganisms; Leibniz Institute DSMZ—German Collection of Microorganisms and Cell Cultures) and Molecular Biotechnology and Microbiology Department (MBMD) collection of fungi (Gdańsk University of Technology, Gdańsk, Poland). Identification of all MBMD isolates was performed by observation of macro- and micromorphology and then confirmed by sequencing of the ITS region, as described by White et al. [45]. Moreover, in case of *Alternaria* spp., *Aspergillus* spp. and *Scopulariopsis* spp. MBMD isolates β-tubulin gene sequencing was performed, as described by Glass and Donaldson [46].

**Table 1:** Organisms used in the study

Organism	Collection
Moulds	
*Scopulariopsis acremonium*	DSM 1987
*S. asperula*	CBS 298.67
	IHEM 2546
*S. brevicaulis*	CBS 112377; CBS 119550; CBS 118474; CBS 340.39
	MBMD (human-derived isolate): W1; MBMD (dog-derived isolate): 19P; MBMD (rabbit-derived isolates): F9; F10
*S. brumptii* (now *Fuscoannellis carbonaria*)	CBS 121662
*S. brumptii* (now *Microascus paisii*)	CBS 116060
*S. canadensis*	CBS 204.61
*S. carbonaria* (now *F. carbonaria*)	CBS 205.61
*S. chartarum* (now *M. chartarus*)	CBS 294.52
*S. chartarum* (now *M. paisii*)	CBS 670.74
*S. coprophila*	CBS 433.83
*S. flava*	CBS 207.61
*S. fusca* (now *S. asperula*)	CBS 117767
	IHEM 14552; IHEM 25912
*S. gracilis* (now *M. gracilis*)	CBS 369.70
*S. koningii* (now *S. brevicaulis*)	CBS 289.38
*S. murina* (now *M. murinus*)	CBS 830.70; CBS 621.70; CBS 864.71
*S. parva*	CBS 209.61; CBS 271.76
*Microascus albonigrescens*	CBS 313.71; CBS 109.69
*M. cinereus*	CBS 664.71; CBS 195.61
	IHEM 25417
*M. cinereus* (now *M. gracilis*)	CBS 116059;
*M. cirrosus*	CBS 116405; CBS 277.34
*M. cirrosus* (now *M. pseudolongirostris*)	CBS 462.97;
*M. longirostris*	CBS 415.64; CBS 196.61
*M. manginii* (now *S. macurae*)	CBS 506.66
*M. manginii* (now *S. candida*)	CBS 132.78
*M. senegalensis*	CBS 594.78
*M. singularis*	CBS 505.66
*M. stoveri* (now *Pithoascus stoveri*)	CBS 176.71
*M. trigonosporus var. terreus* (now *M. terreus*)	CBS 601.67
*M. trigonosporus var.macrosporus* (now *M. macrosporus*)	CBS 662.71
*M. trigonosporus var. trigonosporus* (now *M. alveolaris)*	CBS 494.70
*Acremonium charticola*	MBMD (environmental isolates)
*Acremonium kiliense* (now *Sarocaldium kiliense*)	
*Acremonium strictum* (now *Sarocaldium strictum)*	
*Alternaria alternata*	
*A. brassicae*	
*A. tenuissima*	
*Alternaria* sp.	
*Aspergillus clavatus*	
*A. flavus*	
*A. fumigatus*	
*A. nidulans*	
*A. niger*	
*A. versicolor*	
*Cladosporium cladosporioides*	
*C. herbarum*	
*C. macrocarpum*	
*Fusarium culmorum*	
*F. discolor*	
*F. oxysporum*	
*F. proliferatum*	
*Fusarium solani* (now *Neocosmospora solani)*	
*Mucor racemosus*	
*M. circinelloides*	
*Ochrocladosporium elatum*	
*Penicillium chrysogenum*	
*P. carneum*	
*P. chrysogenum*	
*P. commune*	
*P. crustosum*	
*P. digitatum*	
*P. glabrum*	
*P. hirsutum*	
*P. italicum*	
*P. melinii*	
*P. paneum*	
*P. polonicum*	
*P. verrucosum*	
*Penicillium* sp.	
*Phoma herbarum*	
*Pleospora papaveracea*	
*Rhizopus oligosporus*	
*R. oryzae*	
*Trichoderma viride*	
*Ulocladium chartarum*	
*U. tuberculatum*	
Dermatophytes	
*Epidermophyton floccosum*	MBMD (human-derived isolates)
*Microsporum audouinii*	
*M. canis*	
*M. gypseum*	
*M. nanum*	
*M. persicolor*	
*Trichophyton equinum*	
*T. erinacei*	
*T. interdigitale*	
*T. mentagrophytes*	
*T. rubrum*	
*T. schoenleinii*	
*T. soudanense*	
*T. terrestre*	
*T. tonsurans*	
*T. verrucosum*	
*T. violaceum*	
Yeast-like fungi	
*Candida albicans*	MBMD (human-derived isolates)
*C. catenulata*	
*C. glabrata*	
*C. guillermondii*	
*C. kefyr*	
*C. krusei*	
*C. magnoliae*	
*C. parapsilosis*	
*C. tropicalis*	
*C. utilis*	
*Geotrichum* sp.	MBMD (environmental isolates)
*Rhodotorula mucilaginosa*	
*Saccharomyces cerevisiae*	
Human	MBMD

### DNA Extraction

Isolates were cultured on Sabouraud glucose agar (Biomerieux, Marcy l’Etoile, France) and incubated for up to 14 days at room temperature. DNA from fungal samples (pieces of mycelium of 3–5 mm diameter) was extracted by a 10-min incubation of the sample in 100 µl of extraction buffer (60 mM sodium bicarbonate [NaHCO_3_], 250 mM potassium chloride [KCl] and 50 mM Tris, pH 9.5) in 95 °C and subsequent addition of 100 µl anti-inhibition buffer (2 % bovine serum albumin). After vortex mixing, this DNA-containing solution was used for PCR [47]. All reagents for DNA extraction were purchased from Sigma-Aldrich (Seelze, Germany).

### PCR and Real-Time PCR Assays

On the basis of alignment (VectorNTI; InforMax, Inc.) of β-tubulin gene (*TUBB*) sequences deposited in the NCBI nucleotide database, *Microascus/Scopulariopsis*-specific primers ScopFor (5′ CATCTCGGGCGAGCACGGTC 3′) and ScopRev (5′ CCAGGACAGCACGGGGAACAT 3′) were designed. Primers were then synthesised by Genomed (Warsaw, Poland). PCR mixtures, of 20 µl each, consisted of 10 µl of 2× PCR Master Mix Plus High GC (A&A Biotechnology, Gdynia, Poland), 0.1 µl of each primer (ScopFor, ScopRev) at 100 µM, and 2 µl of DNA. PCR was performed in a 5345 Mastercycler ep gradient S (Eppendorf, Hamburg, Germany). The time–temperature profile included initial denaturation for 3 min at 94 °C followed by 35 cycles of 30 s at 94 °C, 30 s at 68 °C, and 30 s at 72 °C. The presence of specific 285-bp amplicons was examined electrophoretically on a 2 % agarose gel, stained with ethidium bromide.

*S. brevicaulis*-specific PCR assay was performed the same way as previously described [48].

Real-time PCR mixtures, of 20 µl each, consisted of 10 µl of 2× PCR Master Mix SYBR A (A&A Biotechnology, Poland), 0.1 µl of each primer (ScopFor, ScopRev in *Microascus/Scopulariopsis*-specific assay; SbFor, SbRev in *S. brevicaulis*-specific assay) at 100 µM, and 2 µl of DNA. PCR was performed in a LightCycler^®^ Nano Instrument (Roche, Basel, Switzerland). The cycling conditions in *Microascus/Scopulariopsis*-specific assay included an initial denaturation for 3 min at 95 °C followed by 40 cycles of 15 s at 94 °C, 15 s at 68 °C and 30 s at 72 °C. The time–temperature profile in *S. brevicaulis*-specific assay started with initial denaturation for 3 min at 94 °C followed by 40 cycles of 10 s at 94 °C, 10 s at 60 °C and 15 s at 72 °C. The presence of specific amplicons was examined upon melting temperature analysis (80 °C to 95 °C at 0.1 °C/s ramp rate), which followed cycling.

## Results

### *Microascus/Scopulariopsis*-Specific PCR and Real-Time PCR Assay Results

**Fig. 1:**
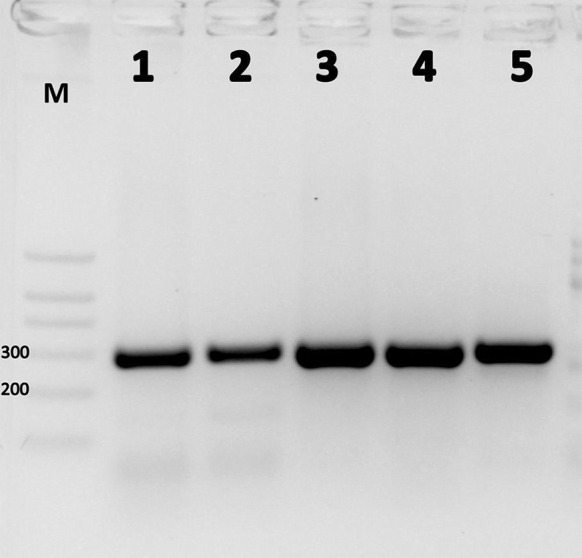
Example of *Scopulariopsis/Microascus*-specific PCR product analysis. *M* molecular size marker (fragment sizes 700, 500, 400, 300, 200 and 100 bp); results of *Scopulariopsis/Microascus*-specific PCR performed for *S. asperula* CBS 298.67 (*lane 1*); *S. brevicaulis* CBS 112377 (*lane 2*); *S. flava* CBS 207.61 (*lane 3*); *S. fusca* IHEM 14552 (*lane 4*); *M. cinereus* CBS 195.61 (*lane 5*)

A 285-bp PCR product corresponding to *Scopulariopsis/Microascus* was observed for all 48 *Scopulariopsis* and *Microascus* spp. DNA samples. No PCR products were detected for 76 other mould isolates, 65 dermatophyte isolates, 30 yeast-like isolates or human DNA (100 % sensitivity and 100 % specificity) (Fig. 1).

**Fig. 2:**
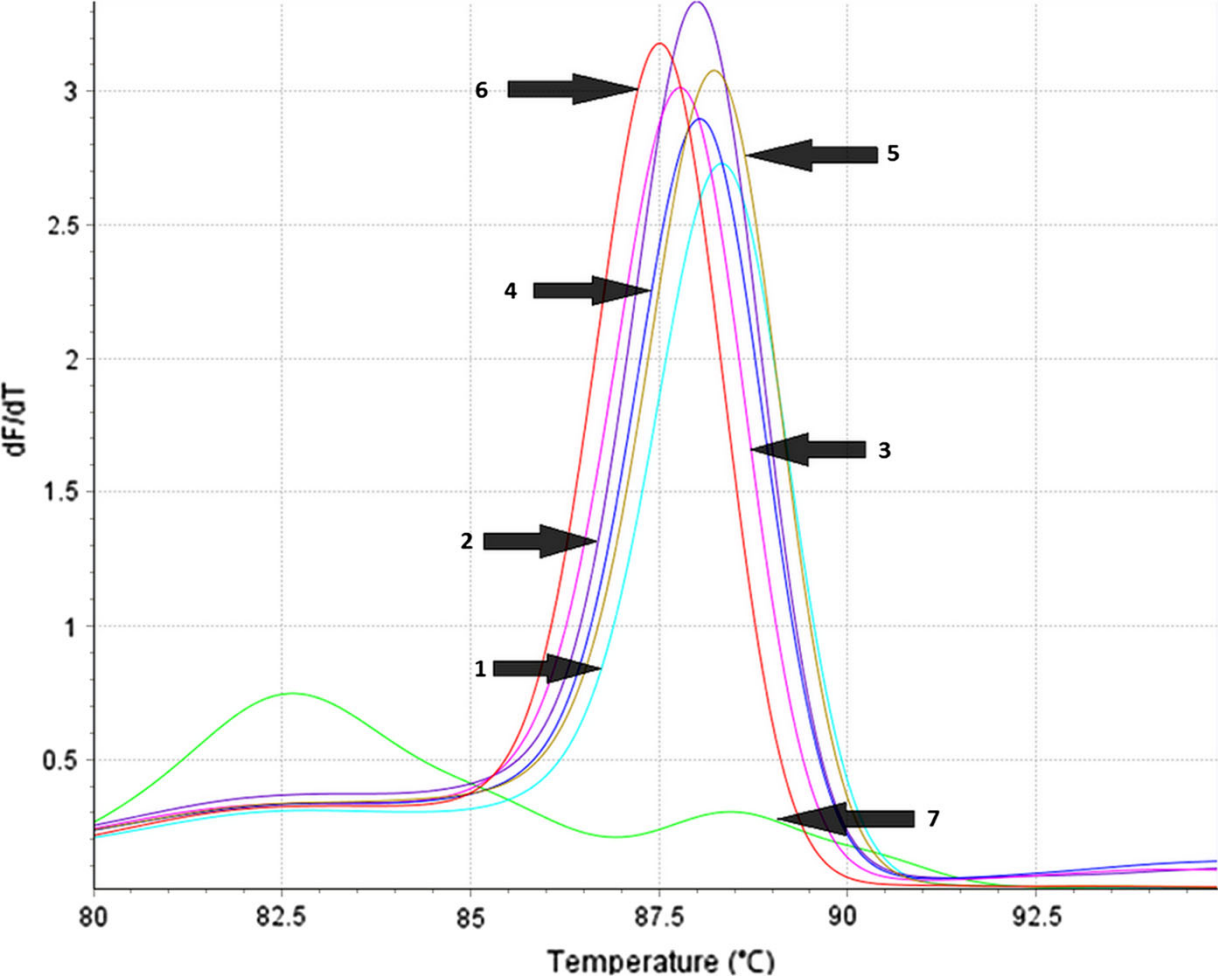
Example of *Scopulariopsis/Microascus*-specific real-time PCR product melting temperature analysis performed for *S. asperula* CBS 298.67 (*1*); *S. brumptii* CBS 121662 (*2*); *S.* *brevicaulis* CBS 119550 (*3*); *S. flava* CBS 207.61 (*4*); *S. fusca* CBS 117787 (5); *M. manginii* CBS 195.61 (*6*); negative control (*7*)

Similar results were obtained when real-time PCR was applied, as amplicon of *T*_m_ range of 87.03–89.02 °C (*C*_t_ = 25.12 ± 4.28), corresponding to *Scopulariopsis/Microascus* spp., was observed only for 48 *Scopulariopsis* and *Microascus* spp. DNA samples and not for any other fungal or human DNA samples (Fig. 2).

### *S. brevicaulis*-Specific PCR and Real-Time PCR Assay Results

**Fig. 3:**
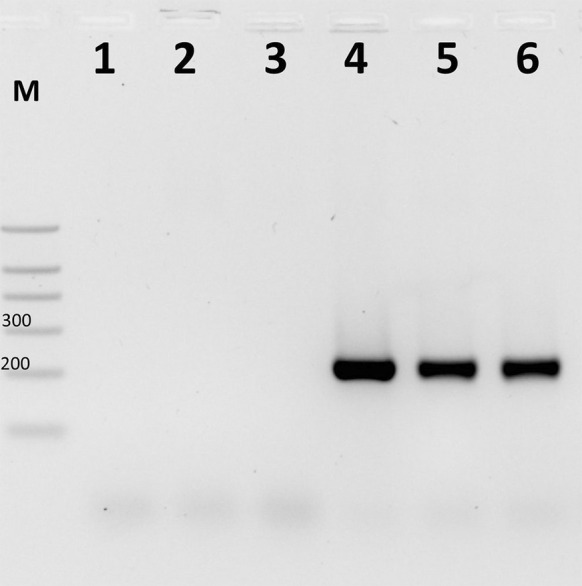
Example of *S. brevicaulis*-specific PCR product analysis. *M* molecular size marker (fragment sizes 700, 500, 400, 300, 200 and 100 bp); results of *S. brevicaulis*-specific PCR performed for *S. asperula* CBS 298.67 (*lane 1*); *S. fusca* IHEM 14552 (*lane 2*); *S. flava* CBS 207.61 (*lane 3*); *S. brevicaulis* CBS 112377 (*lane 4*); *S. brevicaulis* human-derived isolate MBMD-W1 (*lane 5*); *S. brevicaulis* rabbit-derived isolate MBMD-F9 (*lane 6*)

A 223-bp PCR product corresponding to *S. brevicaulis* was observed for 8/8 *S. brevicaulis* and 1/1 *S. koningii* (syn. *S. brevicaulis*) DNA samples. No PCR products were detected for 20 other *Scopulariopsis* spp. strains, 19 *Microascus* spp. strains, 76 other mould isolates, 65 dermatophyte isolates, 30 yeast-like isolates or one human DNA (100 % sensitivity and 100 % specificity for PCR) (Fig. 3).

**Fig. 4:**
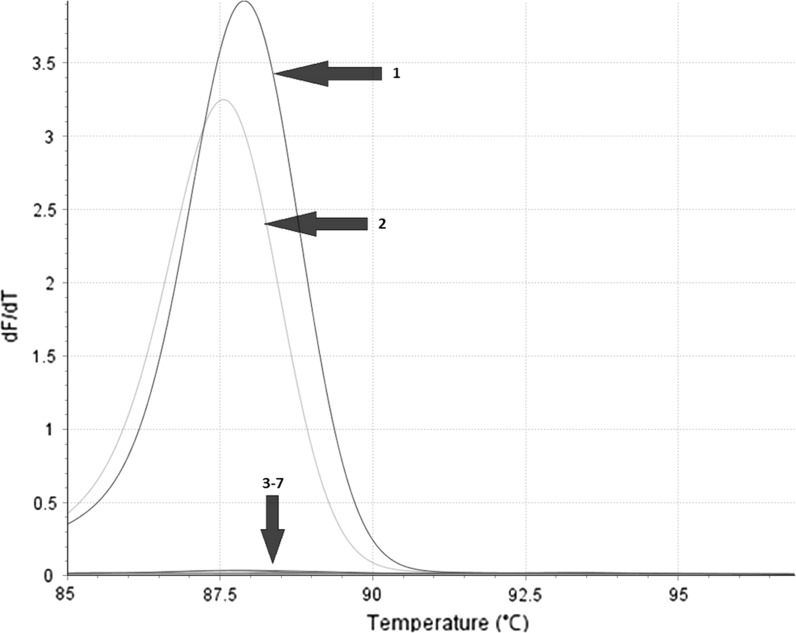
Example of *S. brevicaulis*-specific real-time PCR product melting temperature analysis performed for *Scopulariopsis brevicaulis* CBS 112377 (*1*); *S. brevicaulis* animal-derived isolate MBMD-19P (*2*); *S. asperula* CBS 298.67 (*3*); *S. fusca* IHEM 14552 (*4*); *S. flava* CBS 207.61 (*5*); *M. longirostris* CBS 415.64 (*6*); negative control (*7*)

Accordingly, as a result of real-time PCR, amplicon of *T*_m_ = 87.76 ± 0.20 °C (*C*_t_ = 24.11 ± 4.38) corresponding to *S. brevicaulis* was observed only for 8/8 *S. brevicaulis* and 1/1 *S. koningii* (syn. *S. brevicaulis*) DNA samples and not for any other fungal or human DNA samples (Fig. 4).

## Discussion

At present, identification of pathogenic fungi still largely relies on the evaluation of macro- and micromorphology. Distinction between *Scopulariopsis* and *Microascus* species by using morphological criteria remains useful since the features of conidia and sexual reproductive structures are quite characteristic at the genus level. Two well-recognised disadvantages of these methods, delaying the diagnostic outcome, are the amount of time elapsing from specimen delivery to the diagnostic result acquisition and the requirement of sporulating culture. Diagnosis of disseminated infections is particularly challenging since *Scopulariopsis* fungi are difficult to distinguish from other moulds (e.g. *Aspergillus*, *Fusarium*) upon histopathological examination. Furthermore, the sensitivity of confirmatory blood cultures is poor [44].

Molecular tools have increasingly been adopted in clinical laboratories for the identification of fungi. The sequence analysis of the ribosomal operon has been used for the identification of clinical strains of *Scopulariopsis*, yet the results may not have been fully reliable because of insufficient availability of reference sequences in the public databases [6, 39, 49]. Moreover, the D1/D2 region, the target most frequently used for species identification, exhibits a low interspecific variation in *Scopulariopsis* and *Microascus* genera [6]. Recently, Ropars et al. [50] performed a combined analysis of partial sequences of the large subunit (LSU) rRNA gene, β-tubulin (*TUBB*), and elongation factor 1-α (*EF1*-*α*) genes for the taxonomic circumscription of *Scopulariopsis* species, whereas Bontems et al. [51] developed a PCR–RFLP assay, based on 28S rDNA, for identification of fungi, including *Scopulariopsis* spp., involved in onychomycosis. However, all these methods are laborious and generate rather complicated patterns, thus making them unlikely to be implemented in routine laboratory diagnostics.

All this underlines a need for the development of new methods that would provide simple, rapid and highly specific identification of *Scopulariopsis*/*Microascus* at both genus and species levels. In this study, we present PCR and real-time PCR-based assays that enable genus-specific detection of *Scopulariopsis* spp. and *Microascus* spp. DNA, as well as species-specific detection of *S. brevicaulis* in culture samples. β-Tubulin gene, formerly chosen as one of the targets in phylogenetic studies [50, 52], was confirmed to be an adequate target for genus-specific (*Microascus* spp. and *Scopulariopsis* spp.) and species-specific (*S. brevicaulis*) identification. Developed assays are rapid, easily performed and interpretable, and can serve as useful adjunct tools for the identification of the *Scopulariopsis* spp. and *Microascus* spp. infections. However, further studies are needed to confirm assay’s clinical applicability (sensitivity, direct amplification from various clinical specimens, etc.).

As pointed out by Balajee et al. [53], an increasing number of clinical laboratories begins to assess the usefulness of DNA-based methods for identification of isolates recovered from culture of clinical samples in order to complement morphology-based methods (especially when an isolate displays atypical colour, features, or morphology) or to supplant them when culture results are delayed due to slow or absent sporulation [54]. Moreover, analysis of DNA-based methods results is almost entirely independent from diagnostician experience, and thus, it is easy to implement them in basic laboratories. Precise and timely identification of fungal isolates to species can be extremely important when recovered from high-risk patients, as fungal infections in these patients can be serious, difficult to treat and rapidly fatal [53]. Diagnostic procedures should always be guided by clinical history of the patient and clinician’s suspicion of disease.
